# P290: Infection control in dialysis unit-difficult but not impossible

**DOI:** 10.1186/2047-2994-2-S1-P290

**Published:** 2013-06-20

**Authors:** M Sri Ratnamani, R Rao

**Affiliations:** 1 Microbiology, Apollo hospitals, Hyderabad, India

## Introduction

Infection control in the dialysis unit has its own concerns. These patients are prone to Hospital acquired infections due their lowered immune status, repeated hospital admissions and multiple lines. Infection complications include bacterial and blood borne pathogens either by water, equipment or environment. We implemented a few basic practices to control the infections in our Dialysis unit.

## Objectives

Implementation of Infection control practices in Dialysis unit.

## Methods

First the parameters which needed to be monitored in the Dialysis unit was decided. It included both inpatients and outpatients.

### Quality indicators

1)Number of patients undergoing dialysis developing CLBSI 2)Serconversion in these patients.3)Surveillance of water quality(RO water& Dialysate).

### Implemented practices

a)SUD reuse policy. b)Appropriate handling of dirty hemodialysers. c)Proper disinfection of dialysis machines(internal & External). d)Proper disinfectation of vascular access prior to cannulation. e)Unannounced audits and corrective actions. f)Good & effective Post exposure prophylaxis for staff. g)Dedicated equipment for HBS Ag, HCV positive patients. [1,2]

## Results

By regular monitoring of these parameters we been able to control infections in dialysis patients. This month the dialysis unit got best unit award as a rolling trophy.

## Conclusion

Dialysis units need a stringent Infection control practices. Lack of time and will to implement need to be addressed. Educating the staff and constant motivation helps in achieving this goal. A good staff to patient ratio should also be maintained.

## Competing interests

None declared
